# Conceptual framework for strengthening nurse-initiated management of antiretroviral therapy training and implementation in North West province

**DOI:** 10.4102/hsag.v25i0.1285

**Published:** 2020-02-20

**Authors:** Sheillah H. Mboweni, Lufuno Makhado

**Affiliations:** 1 The Aurum Institute, Moretele Subdistrict, Johannesburg, South Africa; 2 School of Health Science, University of Venda, Thohoyandou, South Africa

**Keywords:** NIMART training, HIV programme, NMART nurse, ART, PHC

## Abstract

**Background:**

The implementation of nurse-initiated management of antiretroviral therapy (NIMART) management training is a challenge in the primary health care (PHC). It is evident from the literature reviewed and the data obtained from the North West province that gaps still exist. There is no conceptual framework providing guidance to NIMART training and implementation.

**Aim:**

Therefore, the aim of this study was to develop a conceptual framework to strengthen NIMART training and implementation in the North West province to improve patients and human immunodeficiency virus (HIV) programme outcomes.

**Setting:**

The study was conducted in the North West Province, South Africa.

**Methods:**

A pragmatic, explanatory, sequential, mixed-methods research design was followed. A descriptive and explorative programme evaluation design was used. Data were collected from two sources: antiretroviral therapy (ART) statistics from District Health Information System (DHIS) & Tier.net of 10 PHC facilities to evaluate and determine the impact of NIMART on the HIV programme and five focus group discussions conducted amongst 28 NIMART nurses and three HIV programme managers to describe challenges influencing NIMART training and implementation.

**Results:**

The study revealed that there was low ART initiation compared to the number of clients who tested HIV-positive. There was poor monitoring of patients on ART, which was evident in the low viral load collection and suppression, high loss to follow-up and deaths related to HIV. Challenges exist and this was confirmed by the qualitative findings, including human resource ratios, training and mentoring and the entire absence of a conceptual framework or model that guides training and implementation.

**Conclusion:**

The study findings were conceptualised to describe and develop a framework needed to facilitate and influence NIMART training and implementation to improve the HIV programme and patient outcomes. Dickoff, James and Wiedenbach’s practice-oriented theory and Donabedian’s structure process outcomes model provided a starting point in the ultimate development of the framework. Although the study was limited to the North West province’s PHC clinics and community health centres and did not include hospitals, it is of high significance as there is no such conceptual framework in the province or in even South Africa.

## Introduction

Human immunodeficiency virus (HIV) and acquired immune deficiency syndrome (AIDS) remain serious global concerns. The prevalence of HIV is increasing even though it has shown a slight decline of 0.8% from 2000, estimated at 19.1%, and meaning that 36.9 million people are living with HIV (PLWH) worldwide UNAIDS (2015). The prevalence varies from country to region. The developing and underdeveloped countries and sub-Saharan region are particularly highly affected, compounded by an increasing burden of tuberculosis (TB) (Joint United Nations Programme on HIV/AIDS [UNAIDS] 2015). The Republic of South Africa (RSA) has the largest epidemic in the world, with an estimated 7 million PLWH at a prevalence rate of 19% amongst adults, of which 13% are amongst men sleeping with men (MSM), 30% pregnant women and 1.4% children. Out of 7 million PLWH, only 60% know their HIV status. However, 3.4 million PLWH can now access antiretroviral therapy (ART) (UNAIDS 2016).

Furthermore, there are 290 000 new HIV infections each year in South Africa and TB incidents stand at 854 per 100 000 cases that include PLWH. Such figures raise serious concerns (UNAIDS 2016). In addition, one-fourth of deaths reported in the RSA are because of HIV-related illnesses and TB has been identified as the leading cause at 8.4% of natural deaths (UNAIDS 2016). World Health Organization’s (WHO) task shifting was implemented for nurses to initiate ART in order to meet this increasing demand for ART since 2009 (WHO 2007). Other policy strategies like 90–90–90 were developed and nurses working in the primary health care (PHC) setting were capacitated to improve the management and control the HIV and TB epidemics (UNAIDS 2014b). Despite these measures, quality health is still a challenge as measured by patient outcomes. This is confirmed by low rates of viral load (VL) suppression, high loss to follow-up (LTFU) and fluctuating numbers of total patients remaining on ART (TROA). In addition, ART initiation amongst HIV-positive antenatal care (ANC) pregnant women, children and TB or HIV co-infected is far below the target of 90% in some districts of the North West province. This is despite the fact that nurses in the province have been trained on HIV management or nurse-initiated management of ART (NIMART) (North West Province Department of Health, 2015–2016). The healthcare system in the North West province continues to experience challenges that have serious repercussions on the implementation of NIMART or HIV management. This fact points to the negative impact on the outcomes of the programmes and patients. Several studies reviewed in this study have revealed that organisational barriers influencing NIMART implementation range from training, human resources (HRs)and budget to patient factors. All these need to be dealt with decisively (Byakika-Kibwika et al. 2015; Davies, Homfray & Venables 2013; Kaposhi, Mqoqi & Schnopflocker 2014; Mbonye et al. 2016; Spies et al. 2016; Zuber et al. 2014). The objective of NIMART training is to produce knowledgeable, skilled and competent nurses who could exhibit confidence as health practitioners. The training also seeks to inculcate positive attitudes in the practitioners as they deal with PLWH. The ultimate stride is that the HIV programme ought to improve patient outcomes (National Department of Health 2011).

However, NIMART nurses still lack confidence to manage PLWH. An initial comprehensive literature review study was conducted, and no framework or model was identified that could guide training and implementation of NIMART. According to Lekhuleni, Kgole and Mbombi (2015), student nurses still lack knowledge and confidence to implement, monitor and evaluate HIV programmes. Furthermore, it was identified that both training and mentoring are partner-driven. Therefore, in this article, the researchers aimed to develop and describe a conceptual framework (CF) that could strengthen NIMART training and implementation to improve patient and HIV programme outcomes in the North West province. A model that characteristically supports the CF should guide effective training and implementation. In this article, the Dickoff, James and Wiedenbach’s (1968) practice-oriented theory and Donabedian’s (1966) structure, process and outcome (SPO) model formed the basis for the development of the CF.

The main objective of this study was to develop and describe a CF for strengthening NIMART training and implementation in the North West province.

## Research methods and design

A mixed-methods paradigm was chosen using a sequential, explanatory, mixed-methods design to obtain an in-depth understanding of the impact of and barriers influencing NIMART training and implementation to produce more complete and well-validated conclusions (Creswell 2009; Munhall 2012). The study was conducted in four phases up until the development of the CF. This included a comprehensive systematic literature review, quantitative programme evaluation and an exploratory descriptive qualitative study.

The CF was crafted based on the findings of QUAN-qual initial studies. Dickoff et al.’s (1968) practice-oriented theory and Donabedian’s (1966) SPO model were used simultaneously to classify and categorise the characteristics, activities and functions of the NIMART training and the implementation of the HIV programme within PHC facilities. There is a symbiotic relationship between Dickoff et al.’s practice-orientated theory and Donabedian’s SPO model. The two therefore provided a starting point for the development of the CF. The typical characteristics, activities and functions were outlined and described through the process of abstraction, starting from the concrete level of experience to the higher level of abstraction in order to determine an ideal framework (Mouton & Marais 1996).

Furthermore, exhaustiveness and mutual exclusiveness were used as criteria to select the most appropriate characteristics best describing the phenomenon by further examining and describing the relationship between the typical characteristics, activities and functions of NIMART training and implementation of the HIV programme (Mouton & Marais 1996). Again, further refinement was performed to eliminate overlapping activities (Table 1).

**FIGURE 1:**
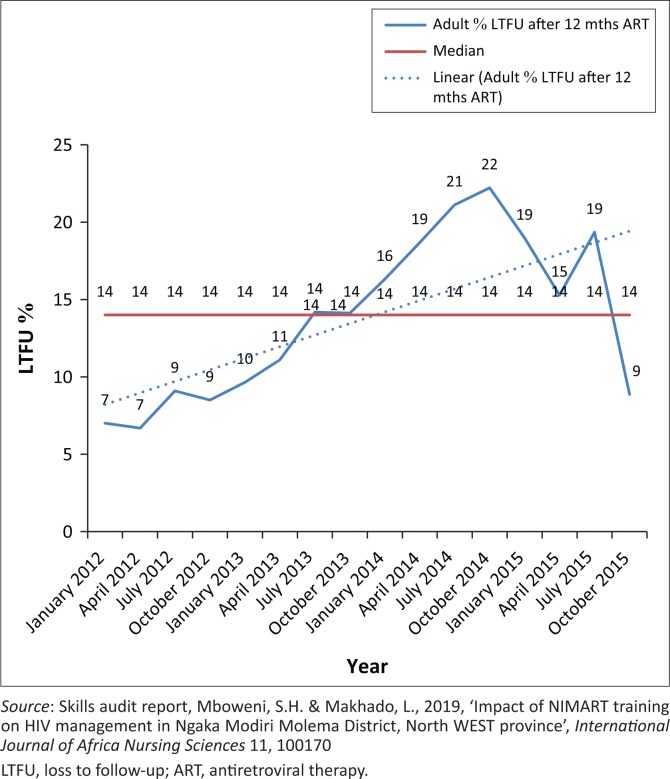
Quarterly percentage of adults’ loss to follow-up after 12 months antiretroviral therapy from January 12 to October 2016, Ngaka Modiri Molema district.

**TABLE 1:** Research designs and method preceding the development of conceptual framework.

Stage	Design	Population	Sampling	Sample size	Context
Phase 1	Systematic literature review	All full text articles	Multimodal	33	-
Phase 2	Descriptive programme evaluation	All NMM district PHC facilities	Simple stratified random	ART Statistics from 10 PHC facilities (five CHCs and five clinics)	Rural NMM
Phase 3	Exploratory descriptive	All NMM NIMART-trained PNs	Non-probability purposive	28	Rural NMM
Phase 4	Dickoff et al.’s (1968) practice-oriented theory and Donabedian’s (1966) SPO model	PHC facilities (clinics and CHCs) NIMART-trained PNs	Simple stratified random and purposive	10 PHC facilities and 28 NIMART PNs	Rural NMM

NIMART, nurse-initiated management of antiretroviral therapy; NMM, Ngaka Modiri Molema; PHC, primary healthcare; CHC, community health centre; PN, professional nurse; SPO, structure, process and outcome.

Stratified simple sampling was used to randomly select PHC facilities from the predominantly rural Ngaka Modiri Molema (NMM) district of the North West province and purposive sampling was used to select NIMART-trained nurses and programme managers directly involved in the management of the TB and HIV programme.

Quantitative data were collected from a secondary source, which includes the District Health Information System (DHIS) and three integrated information electronic registers (Tier.net), from January 2012 to December 2016. The following variables were measured: facility information (facility type, geographical situation, number of professional nurses, number of nurses trained and not trained, number of nurses initiating ART, etc., see Table 2), adults started on ART during this month – naïve; children under 15 years started on ART during this month – naïve; ANC pregnant women initiated on ART and number of professional nurses (PNs) trained on NIMART from regional training centre (RTC) skills audit report (North West province skill audit report 2015). Additional tracer indicators of monitoring VL, VL collection (VLC), VL suppression (VLS), LTFU and death were analysed to obtain a clearer picture of the impact of NIMART training on patients receiving ART in the NMMD. Furthermore, five focus group discussions (FGDs) with 28 NIMART nurses (5, 5, 6, 5 and 7 NIMART nurses per group, respectively)which lasted for 90–120 min and unstructured in-depth interviews amongst HIV programme managers were used to collect data in the qualitative study. Data saturation was reached with 28 NIMART nurses (De Vos et al. 2012).

**FIGURE 2:**
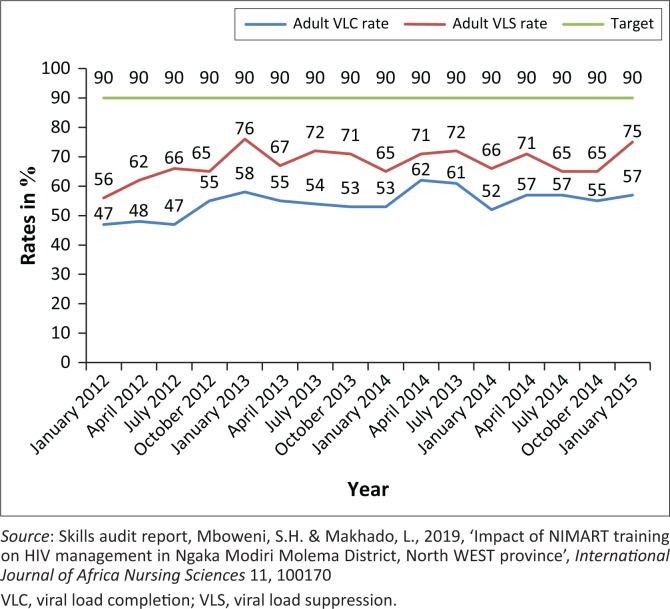
Adult viral load completion and viral load suppression rate at 12 months, January 12 – January 15, Ngaka Modiri Molema district.

**TABLE 2:** Nurse-initiated management of antiretroviral therapy training coverage in Ngaka Modiri Molema Regional Training Centre, 2016.

Sub-district	Area	Facilities	PNs in the facility	Trained		Not trained		Initiating ART		Certificated		Not certificated	
				*n*	%	*n*	%	*n*	%	*n*	%	*n*	%
1	U	CHC 1	17	13	76.0	4	23.5	13	0.0	9	0.0	4	30.7
	R	Clinic 1	13	10	77.0	3	23.0	10	0.0	7	0.0	3	30.0
2	R	CHC 2	15	14	93.0	1	6.6	14	0.0	11	0.0	3	21.4
	R	Clinic 2	2	2	100.0	0	-	2	0.0	0	0.0	2	100.0
3	SU	CHC 3	12	9	75.0	3	25.0	9	0.0	3	0.0	6	66.6
	R	Clinic 3	4	3	75.0	1	25.0	3	0.0	0	0.0	3	100.0
4	SU	CHC 4	8	6	75.0	2	25.0	6	0.0	5	0.0	1	16.6
	R	Clinic 4	2	2	100.0	0	-	2	0.0	1	0.0	1	50.0
5	SU	CHC 5	17	10	58.8	7	41.0	10	0.0	4	0.0	6	60.0
	R	Clinic 5	1	1	100.0	0	-	1	0.0	0	0.0	1	100.0

		**Total**	**91**	**70**	**76.9**	**21**	**23.0**	**70**	**100.0**	**40**	**57.1**	**30**	**42.8**

*Source*: Skills audit report, Mboweni, S.H. & Makhado, L., 2019, ‘Impact of NIMART training on HIV management in Ngaka Modiri Molema District, North WEST province’, *International Journal of Africa Nursing Sciences* 11, 100170

R, rural; SU, semi-urban; U, urban; ART, antiretroviral therapy; CHC, community health centre; PN, professional nurse.

Data from DHIS were extracted as pivot tables into Excel spread sheet, analysed and presented quantitatively in tables and graphs using descriptive statistics. Atlas.ti software was used to analyse the qualitative data supported by the basic steps of descriptive analysis to analyse qualitative data: coding, analysis and description (Friese 2012). The researcher read and re-read the transcribed data until themes emerged. Themes were then grouped into codes according to the similarity and later classified into categories.

Trustworthiness of the study findings was enhanced using mixed-methods design, validating data with participants and keeping all tape-recorded data and notes safe for future reference. The researcher also spent enough time with participants until data saturation, which made possible to scrutinise and amplify the data. An initial pilot study facilitated the development and refinement of study methods.

## Ethical consideration

The study received approval from the Ethics and Research Committee of the North-West University (approval number: NWU-00607-17-A9) and permission was granted by the NW Department of Health. Voluntary, written informed consent was obtained from all the participants and they were informed of their rights to withdraw from the study at any time. The data collected were recorded after permission was granted and were kept safe and locked. Privacy, anonymity and confidentiality in all procedures were maintained in the spirit of ethical conduct.

## Results

Meta-inference and interpretation of the quantitative and qualitative results were conducted to supplement the quantitative findings, and these are presented in the following:

### Nurse-initiated management of antiretroviral therapy training coverage in the primary health care facilities and antiretroviral therapy intake constraints

The NIMART training coverage is herein referred to as the total number of professional nurses trained in NIMART out of the total number of professional nurses in that facility. The quantitative findings revealed that all PHC clinics from rural, urban and semi-urban areas (100%) are within the set coverage target of 75% and only one Community Health Centre (CHC) from a semi-urban area (58.8%) is below the target. About 23% of PNs were still not trained and from those trained, all had embarked on initiating ART (Table 2). The remaining 42.8% of PNs had not been assessed and certificated for competency.

The qualitative results revealed that newly qualified PNs from the nursing colleges and university reported that they were frustrated by not knowing what to do and tended to transfer patients who tested positive to another facility or book them for another day. One NIMART nurse stated that ‘[*I*] wish NIMART can be introduced in the college or university curriculum so that I know how to manage TB/HIV patients before I join the Department [*of Health*]’ (P13, FGD3, female) and another nurse indicated that ‘I was so disappointed and frustrated at the same time for failing to help the client’ (emotional, raising the voice) (P8, FGD2, male). Such transfer of responsibilities could negatively contribute to low ART initiation and losing patients before initiation. The study revealed that not all HIV-positive patients are initiated on ART in both PHC clinics and CHCs, especially amongst children and ANC pregnant women, despite the introduction of the prevention of mother-to-child transmission (PMTCT)policy in 2013 (National Department of Health 2014) and universal test and treat in September 2016 (National Department of Health 2016) (Tables 2 and 3). These policies specifically direct that all patients who are HIV-positive, including pregnant women, should be initiated on ART without considering the Cluster of Differentiation 4 cell count.

**TABLE 3:** HIV testing versus antiretroviral therapy initiation in Ngaka Modiri Molema district primary health care and community health centre.

Sub-district	Facilities	HTS all ages	January – December 2012	January – December 2013	January – December 2014	January – December 2015	January – December 2016	Total	
								*n*	%
1	CHC 1	HIV tested	3177	3765	4122	4723	4589	20 376	-
		HIV +ve	419	392	449	399	359	2018	-
		Total initiated	259	250	326	221	258	1314	68
		Yield	13	10	11	8	8	51	-
2	CHC 2	HIV tested	1111	1673	1723	1803	2211	8521	-
		HIV +ve	239	263	299	194	201	1196	-
		Total initiated	187	136	191	96	103	713	60
		Yield	22	16	17	11	9	74	-
3	CHC 3	HIV tested	749	603	849	895	501	3597	-
		HIV +ve	219	189	209	192	258	1067	-
		Total initiated	109	122	127	135	34	527	49
		yield	29	31	25	21	51	158	-
4	CHC 4	HIV tested	503	488	591	607	678	2867	-
		HIV +ve	179	191	122	104	111	707	-
		Total initiated	99	139	92	124	34	488	69
		yield	36	39	21	17	16	129	-
5	CHC 5	HIV tested	1122	1599	1879	2088	2322	9010	-
		HIV +ve	199	319	352	409	489	1768	-
		Total initiated	233	248	205	152	212	1050	59
		yield	18	20	19	20	21	97	-

Source: Skills audit report, Mboweni, S.H. & Makhado, L., 2019, ‘Impact of NIMART training on HIV management in Ngaka Modiri Molema District, North WEST province’, International Journal of Africa Nursing Sciences 11, 100170

+ve, positive; HIV, human immunodeficiency virus; HTS, HIV Testing Services; CHC, Community Health Centre.

This was found to be closely linked to the qualitative results (Table 5), showing clearly that NIMART nurses are not initiating ART because they are not trained. The other reason lies in the fact that some PNs have negative attitudes towards the HIV programme. There is also evidence that inadequate in-service training on updated guidelines, poor compliance to guidelines, lack of confidence, especially to manage children and TB and HIV co-infected patients, and poor linkage to care within the facility only serve to exacerbate the situation with regard to PLWH. In addition, NIMART nurses revealed that data quality and management are poor. This contributes to low ART initiation and frustrates them, as their hard work is not recognised. The study also disclosed that NIMART nurses fail to complete and audit clinical records and such practices compromise the quality, capturing and reporting of critical data.

The study further revealed that patients contribute to the low ART initiation, as facilities lose them before they are initiated. Linkage of HIV-positive clients to treatment is far below the target of 90% and this has a negative impact on the achievement of the ambitious target of 90–90–90 strategy by 2020. It was revealed that patients still suffer from stigma and discrimination and do not disclose their status to their partners and families. Such non-disclosure is bound to affect their children. The poor socio-economic status faced by PLWH is also a barrier that massively adds to non-adherence to appointments as they move from one area to another looking for jobs. In addition, the study results also revealed that the HIV positivity yield is fluctuating in all selected PHC facilities.

The study findings revealed several challenges influencing NIMART implementation (Table 4). Nurse-initiated management of antiretroviral therapy -trained nurses reported that the healthcare organisation negatively influenced the implementation of NIMART training. Furthermore, they revealed that the PHC infrastructure was not well maintained and the facilities were also too small to cater for the increasing number of patients who wished to access PHC, including HIV services. Consequently, this could expose both staff and patients to cross-infection because of overcrowding.

**TABLE 4:** HIV testing versus antiretroviral therapy initiation in Ngaka Modiri Molema district community health centre and physical healthcare clinics.

Sub-district	Facilities	HTS all ages	January – December 2012	January – December 2013	January – December 2014	January – December 2015	January – December 2016	Total	
								*n*	%
1	Clinic 1	HIV tested	289	292	282	345	339	1547	-
		HIV +ve	51	35	47	38	47	218	-
		Total initiated	35	17	29	27	32	140	64
		yield in %	18	12	17	11	14	71	-
2	Clinic 2	HIV tested	399	273	297	304	312	1585	-
		HIV +ve	59	55	53	59	49	275	-
		Total initiated	41	19	42	45	40	187	68
		yield in %	15	20	18	19	16	88	-
3	Clinic 3	HIV tested	117	121	133	191	189	751	-
		HIV +ve	47	43	37	29	34	190	-
		Total initiated	32	35	23	8	0	98	51
		yield in %	40	36	28	15	18	137	-
4	Clinic 4	HIV tested	300	297	201	199	320	1317	-
		HIV +ve	65	46	71	66	88	336	-
		Total initiated	37	44	40	45	47	213	63
		yield in %	22	15	35	33	28	133	-
5	Clinic 5	HIV tested	1167	1357	1978	1859	1972	8333	-
		HIV +ve	304	340	297	202	301	1444	-
		Total initiated	210	209	146	136	146	847	59
		yield in %	26	25	15	11	15	92	-

+ve, positive; HIV, human immunodeficiency virus; HTS, HIV testing services; CHC, community health centre.

Some patients were reported to wait outside and felt uncomfortable when they had to wait for long times before they were attended to. The study revealed that there is a critical shortage of skilled HRs and experienced nurses are resigning. In addition, NIMART nurses revealed that the Department of Health always argued that budget constraints were the main reason why vacancies were not filled. This was also highlighted through the reported high staff turnover, especially amongst skilled NIMART nurses. The remaining NIMART nurses and programme managers reported that they are overworked, and this puts them under severe pressure. Because the NIMART-trained nurses are stressed, some display negative attitudes towards the HIV programme and PLWH. These also contribute to poor compliance to guidelines and patients having to wait for long hours for care, while some are left unattended. Both NIMART nurses and programme managers revealed that there is no support, coaching and supervision from management and the absence of all these demoralises them (Table 5).

**TABLE 5:** Thematic analysis of challenges influencing nurse-initiated management of antiretroviral therapy training and implementation.

Number	Theme	Categories	Subcategories
1	NIMART training	1.1. Lack of NIMART-standardised curriculum	1.1.1. Poor integration of theory and practice
		1.2. Lack of involvement of training, education and practice stakeholder in NIMART training and implementation	1.2.1. Lack of involvement of quality assurance bodies, nursing colleges and universities
			1.2.2. Lack of pre-service training
		1.3. Inadequate continued development	1.3.1. Inadequate continuous in-service
		1.4. Lack of training model or conceptual framework	1.4.1. Lack of guidance to NIMART training and implementation
2	Healthcare system	2.1. Patient-related challenges	2.1.1. Socio-economic status and adherence
			2.1.2. Lack of disclosure
		2.2. Human resource challenges	2.2.1. Shortage of skilled healthcare workers
			2.2.2. Staff attitude regarding HIV management
			2.2.3. Lack of confidence
			2.3.1. Poor adherence to data management SOPs
			2.3.2. Lack of data verification
			2.3.3. Incomplete clinical records
			2.3.4. Inconsistent clinical records quality audits
		2.4. Poor infrastructure	2.4.1. Poorly maintained PHC facilities
			2.4.2. Overcrowding
			2.5.1. Lack of district mentoring strategy
			2.5.2. Lack of support systems from management
			2.5.3. Poor compliance to policies and guidelines

NIMART, nurse-initiated management of antiretroviral therapy; PHC, physical healthcare; HIV, human immunodeficiency virus; SOP, structure, process and outcome.

### Nurse-initiated management of antiretroviral therapy training challenges

In addition to the healthcare organisation and patient factors that have been discussed, the study revealed flaws in NIMART training and mentoring. These flaws influence implementation as indicated in Table 4.

Training and mentoring are partner-driven and there are no standard curriculum and mentoring strategy.

Training is conducted differently over 5 or 10 days. Community service nurses left the higher education institution (HEI) without the basic knowledge and skills on ART or HIV management; hence, some patients are turned away and advised to come back when there is a NIMART nurse. If this does not happen, the reports indicate that patients are transferred to another PHC facility or hospital. These factors were reported to frustrate community service nurses and weaken linkages to healthcare. Another aspect revealed in this study is the use of unskilled facilitators and ineffective traditional training strategies that lack integration of theory and practice and thus result in poor learning outcomes. The study revealed that NIMART training is not recognised or registered with the regulatory bodies like South African Nursing Council (SANC) or health and welfare sector education and training authority (HWSETA) for quality and is not regarded as part of continuous professional development (CPD) to keep nurses and lecturers updated on HIV management.

### Additional tracer indicators to monitor quality of antiretroviral therapy management

Figures 1 and 2 illustrate analyses of monitoring of VL completion, viral suppression and LTFU rate from Tier.net to obtain a clearer picture of the impact of NIMART training on patients receiving ART.

### Instability of total patients remaining on antiretroviral therapy: Poor adherence and retention to care

The quantitative results revealed fluid and fluctuating patterns in TROA. This was also confirmed by the fluctuation in adults’ LTFU after 12 months of starting ART at an average of 14% and low ART initiation amongst patients who tested positive or eligible for ART (Tables 1 and 2). This is closely linked with the qualitative findings, which include a combination of patients’ psychosocial and economic factors, NIMART nurses’ level of training and healthcare system challenges.

### Poor monitoring of treatment outcomes

The study revealed that even though access to ART has increased, the quality of HIV services for PLWH is unsatisfactory. This is evident in the poor monitoring of VL rate at 12 months after starting ART, which is at an average of 54% low and viral suppression at 56%, which is far below the target of 90%. This confirms the qualitative results that NIMART nurses are not compliant to guidelines despite training.

### Conceptual framework to strengthen nurse-initiated management of antiretroviral therapy training and implementation

The CF was developed based on Dickoff et al.’s (1968) practice-oriented theory and Donabedian’s (1966) SPO model. Both Dickoff et al.’s practice-oriented theory and Donabedian’s SPO model enabled the researcher to identify and incorporate the different features so that these coalesce to provide the emerging phases 1–3 results, and eventually a CF. The development of CF is described next.

### Expected persons to implement the nurse-initiated management of antiretroviral therapy training conceptual framework (the agent)

According to Dickoff et al. (1968), an agent refers to a person or thing that implements the framework. According to Donabedian’s (1966) SPO model, this refers to the structure that enables the implementation of the CF, including the recipient and the context. In this study, the national, provincial and district-level RTCs, and developmental partners including SANC and HEI are the chief implementers of the framework. It is evident from the study findings that they lack a CF to guide and strengthen the implementation of NIMART training that strives to achieve the intended goal of a skilled, confident, competent NIMART nurse cohort that renders quality HIV services and improves patient outcomes. In most instances, it does not address the gaps affecting the HIV programme.

### The recipient of nurse-initiated management of antiretroviral therapy training (recipient)

According to Dickoff et al. (1968), a recipient is a person or thing that receives action from the agent. In this framework, the recipient is any healthcare worker receiving NIMART training. These can be professional nurses, facilitators, student nurses, nurse educators or programme managers. According to WHO, task shifting was recommended for nurses to initiate and manage ART rather than rely solely on the doctors to meet the increasing demand of healthcare services. It is evident from the study results that the agent should provide comprehensive quality training to improve the skills, competence and confidence of NIMART nurses in the provision of quality care to the patients and to facilitators and educators to transfer the skills to these students. In addition, the patients indirectly benefit from the NIMART training as they equally receive care from the NIMART nurse.

### The context where nurse-initiated management of antiretroviral therapy training can be implemented

Context refers to resources, activities and environment which enable or facilitate implementation (Dickoff et al. 1968). It is evident from the study that a combination of organisational resources and a conducive, safe and comfortable environment within the district’s PHC system can facilitate implementation. The availability of adequate, independent, experienced, skilled HR with positive attitudes towards PLWH and HIV programme facilitates robust implementation. Again, the development of a standard integrated NIMART curriculum and the use of effective interactive strategies that stimulate critical thinking and facilitate integration of theory and practice can influence implementation. Moreover, provision of NIMART or HIV management pre-service training to student nurses, CPD and in-service training on HIV changes can facilitate NIMART implementation. It is evident from the study that availability of good communication and relationship skills, including compliance to HIV and TB and PHC policies, guidelines, protocols and SOPs, facilitates implementation. In addition, treating patients and nurses with respect and attending to their concerns or challenges can also facilitate implementation.

Furthermore, maintenance of the physical infrastructure of the PHC facilities, having enough space, reorganisation of the facility in line with the ideal clinic’s Integrated Clinical Services Management (ICSM) standards and reducing waiting times can facilitate implementation and quality. Prioritising the PHC in budget planning is necessary to deal with the overt challenges influencing implementation. Provincial and district management team support, flexibility, coaching and supervision are all necessary to influence and facilitate discipline and meaningful implementation.

### The support system to improve nurse-initiated management of antiretroviral therapy implementation (dynamics)

Dynamics refer to the sources of power or energy amongst the activities (Dickoff et al. 1968). According to Donabedian (1966), this refers to the process that facilitates the implementation of the CF, including the guiding principles. It is evident from the study findings that motivation, acknowledgement and recognition of NIMART nurses for rendering services under difficult conditions substantially influence and facilitate implementation. Intrinsic and extrinsic recognition is necessary to motivate and enhance the performance. This would invariably boost their self-esteem, build confidence and improve the sense of responsibility and feeling worthy to the department. Again, the Department of Health should meet NIMART nurses’ needs and deal with their frustrations. Furthermore, avoiding negative criticism and blame–punishment feedback would exert a great influence on successful implementation.

### The guiding procedure or rule (principle or process)

Guiding principle refers to the rule, technique, protocol and routine governing the activities to achieve the terminus (Dickoff et al. 1968). It is evident from the study results that quality training, mentoring, support and compliance to policies, guidelines, SOPs and protocols are the guiding principles that facilitate achievement of patient and framework outcomes. Again, monitoring, reporting and evaluation facilitate identification of gaps, signs of danger and success to arrive at the terminus. Another principle to facilitate and influence implementation is involvement of all internal and external stakeholders, which includes district clinical specialist teams (DCSTs), facilitators, RTC managers and the province and district leadership developmental partners, HEI, SANC and HWSETA in implementation.

### The outcome of implementation of nurse-initiated management of antiretroviral therapy training conceptual framework (terminus)

Terminus refers to the outcomes or results of an activity (Dickoff et al. 1968). Donabedian (1966) describes a terminus as the product or outcomes of the structure and process. It is evident from the study findings that the outcomes of effective and efficient implementation of NIMART training facilitate the production of confident, competent and skilled NIMART nurses, who are compliant to policies and guidelines. This will facilitate the improvement of patient health status by increasing linkages to ART, improving adherence and retention to care, low LTFU, viral suppression and decanting of stable patients and relieving pressure on NIMART nurses. Furthermore, this facilitates the reduction of death rate and increases life expectancy.

**FIGURE 3:**
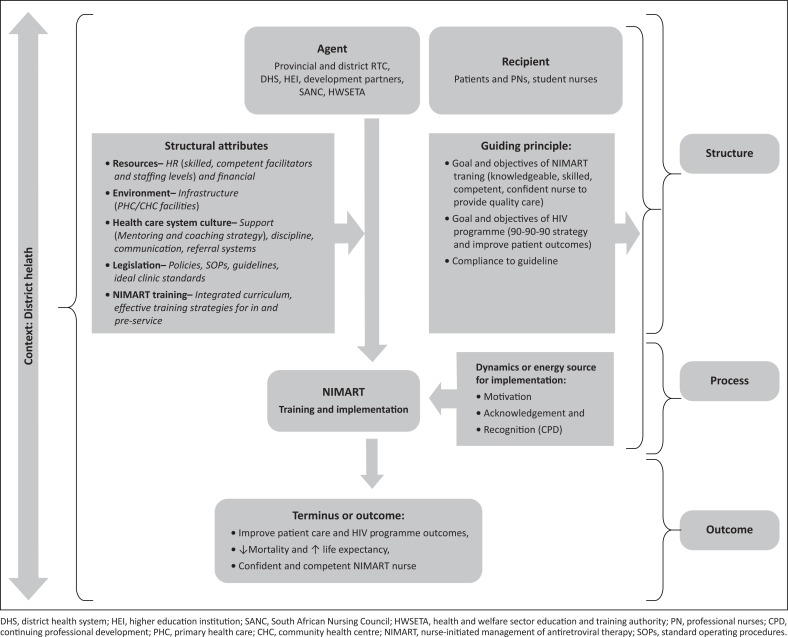
Conceptual framework for strengthening nurse-initiated management of antiretroviral therapy training and HIV management implementation.

Dickoff et al.’s (1968) six survey list and Donabedian’s (1966) SPO model were categorised and classified with the characteristics and activities from the study findings to develop a CF that can facilitate and influence improvement of NIMART training and implementation in the North West province (NW) district health system, thus improving the outcomes (Figure 3).

## Discussion

The purpose of this study was to conceptualise the study findings to develop and describe a CF that could provide guidance and strengthen NIMART training and implementation to improve patient and HIV programme outcomes in the North West province. The CF was developed based on Dickoff et al.’s (1968) practice-oriented theory and Donabedian’s (1966) SPO model. The study found that even though access to ART has increased, ART initiation versus HIV-positive patients is low, especially amongst children and ANC (pregnant) women and poses a risk of mother to child transmission, while nurses are trained on NMART. Abuogi, Smith and McFarland (2016) opined that failure to initiate or retain children on ART leads to early mortality. According to Van der Walt, Lancaster and Shean (2016), Anigilaje et al. (2016) and Teklu et al. (2017), early ART initiation reduces transmission of HIV infection, death and incidences of TB. Deconinck et al. (2015) and Adedinsewo et al. (2014) also confirmed that timing of ART initiation also reduces opportunistic infection and improves the health of PLWHV. Reddy et al. (2016), Gesesew et al. (2017) and McNairy et al. (2017) confirmed that poor linkage of HIV-positive patients to care results in early LTFU and delayed ART initiation. Grimsrud et al. (2015) stated that reorganisation of the ART programme and a proper down referral system could reduce LTFU. The universal test and treat policy should be implemented to improve patient outcomes and achieve the ambitious 90–90–90 targets (UNAIDS 2014).

Monitoring of patients on ART is poor and exposes patients to drug resistance, complications and death.

Monitoring assists in early identification of drug interactions, treatment failure and early switching to other drugs or regimens (Cope et al. 2015; Wilhelmson et al. 2016). Life expectancy of PLWH could be improved if LTFU is considered seriously (Patterson et al. 2015). It is evident from the study results that NIMART nurses still lack confidence and competence because of the level of NIMART training and mentoring received, which along with the organisational factors influence implementation (Davies et al. 2013; Mack et al. 2015; Mbonye et al. 2016; Nyasulu et al. 2013; Oladele et al. 2017; Owens & Moroney 2015). It is also evident from the study findings that these barriers should be dealt with decisively to improve outcomes. De La Mata et al. (2017) stated that facility resources contribute to implementation and reduction of LTFU. Nurse-initiated management of antiretroviral therapy nurses did not comply with guidelines and compromised the quality of management provided. It was from these issues that the need originated to develop a CF to strengthen NIMART training and implementation in the North West province.

## Conclusion

Nurse-initiated management of antiretroviral therapy training has significant impact on HIV management as it has resulted in the expansion of the ART programme and increased access to ART services at the PHC level in the North West province.

However, the quality regarding HIV care and management is still a challenge even though PNs are trained on NIMART. It is evident from the study findings that various factors such as healthcare system, patients and training outcomes influence the implementation of NIMART training. The developed CF has the potential to strengthen NIMART training and implementation, thereby promoting positive HIV management outcomes.

### Limitations of the study

This study was limited to one district in the North West province only, focusing on the PHC, and did not include hospitals. Despite these limitations, the study findings are significant as there is no such CF in the province or even in South Africa.

### Practical implications of the study

With reference to the study findings, the CF developed could facilitate improved implementation of NIMART training in the PHC facilities, thus achieving patient and HIV programme outcomes. Again, NIMART training can be improved to produce a skilled, competent and confident nurse cohort who can provide quality HIV and healthcare services. Dealing with challenges influencing NIMART training can facilitate compliance to policies and guidelines, thus improving outcomes.
